# Pulmonary embolization with cyanoacrylate after obliteration of gastric varicose veins

**DOI:** 10.36416/1806-3756/e20240036

**Published:** 2024-06-21

**Authors:** Camila Alcantara Quidigno, Daniela de Lima Guerra, Paulo Henrique Ramos Feitosa

**Affiliations:** 1. Hospital Regional da Asa Norte - HRAN - Brasília (DF) Brasil.

A 46-year-old woman with a history of portal vein and bile duct injury during cholecystectomy reported hematemesis, melena, and syncope. A previous endoscopy showed fine and medium esophageal varices. She was hospitalized with massive melena. A new endoscopy was performed, and hemostasis of bleeding was carried out using cyanoacrylate. The patient achieved clinical stability. Subsequently, the patient developed dyspnea and desaturation. A chest CT showed hyperdense material in structures, such as the gastric fundus, which is compatible with hemostasis material. Chest CT angiography showed hyperdense material in the segmental branch of the middle lobe and in the medial basal subsegmental branch of the right lower lobe, leading to partial filling failure, which is compatible with cyanoacrylate embolization.

Treatment of variceal bleeding with cyanoacrylate in endoscopy is effective in patients with liver disease and can reduce the size of varices on the first application.^1^ However, this procedure can be complicated by embolization of the resulting polymer in some vessels, such as pulmonary arteries,^1^ especially in patients with large varicose veins that require larger volumes of sclerosing agent.^2^ Embolization appears to be more common in patients who received a greater volume of this agent.^3^ Treatment for cyanoacrylate pulmonary embolism is mainly supportive.^2^

[Fig f1]

**Figure 1 f1:**
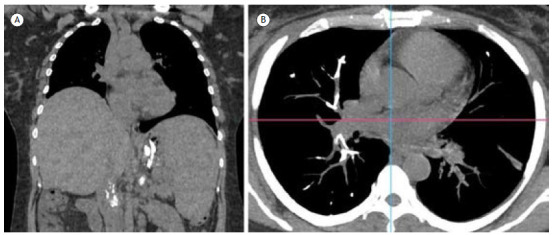
In A, a coronal section of chest CT showing hyperdense material in the topography of the gastric fundus, splenic hilum, left renal vein, and inferior vena cava, which is compatible with hemostasis material. In B, the axial section also showed hyperdense material in the segmental branch of the middle lobe and in the medial basal subsegmental branch of the right lower lobe, leading to partial filling failure, which is compatible with cyanoacrylate pulmonary embolization.
